# Impact of the COVID-19 pandemic on the services provided by the Peruvian health system: an analysis of people with chronic diseases

**DOI:** 10.1038/s41598-024-54275-7

**Published:** 2024-02-13

**Authors:** David Villarreal-Zegarra, Luciana Bellido-Boza, Alfonso Erazo, Max Pariona-Cárdenas, Paul Valdivia-Miranda

**Affiliations:** 1 Intendencia de Invetigación y Desarrollo, Superintendencia Nacional de Salud, Lima, Peru; 2 Instituto Peruano de Orientación Psicológica, Lima, Peru; 3 Escuela de Medicina, Universidad César Vallejo, Trujillo, Peru; 4 Facultad de Ciencias de la Salud, Universidad Peruana de Ciencias Aplicadas, Lima, Peru

**Keywords:** Interrupted time series analysis, Chronic diseases, COVID-19, Peru, Health care, Public health

## Abstract

During the pandemic, many individuals with chronic or infectious diseases other than COVID-19 were unable to receive the care they needed due to the high demand for respiratory care. Our study aims to assess the impact of the COVID-19 pandemic on services provided to people with chronic diseases in Peru from 2016 to 2022. We performed a secondary database analysis of data registered by the comprehensive health insurance (SIS), the intangible solidarity health fund (FISSAL), and private healthcare institutions (EPS), using interrupted time series analysis. Our study identified 21,281,128 individual users who received care. The pooled analysis revealed an average decrease of 1,782,446 in the number of users receiving care in the first month of the pandemic compared with the expected values for that month based on pre-pandemic measurements. In addition, during the pandemic months, there was an average increase of 57,911 in the number of new additional single users who received care per month compared with the previous month. According to the time-series analysis of users receiving care per month based on each chronic disease group, the most significant decreases included people with diabetes without complications and chronic lung disease.

## Introduction

The COVID-19 pandemic occurred against a backdrop of the high prevalence of chronic diseases globally^1^. During the pandemic, people with chronic or infectious diseases other than COVID-19 often had difficulty accessing the care they needed. This was due to the prioritization of treatment for respiratory diseases, mainly COVID-19, which led to overburdened health systems^2^. As a result, the people with chronic diseases were not addressed or included in prevalence reports. For example, according to the cases reported by the Finnish healthcare system, new cancer cases decreased by 5%, and type-2 diabetes cases decreased by more than 16%^3^. This decrease in chronic disease care may be attributed to complications in timely diagnosis and treatment during the pandemic^2^. In addition, the pandemic changed people’s lifestyle^4^ and access to medication^5^. As a result, individuals with pre-existing diseases experienced a 2.6-fold increase in disease severity ^6^, so the need for health insurance for the entire Peruvian population became more evident.

Global public health insurance is crucial to achieving universal health insurance^7^. Public health insurance can reduce access inequalities and ensure quality care among low-income individuals^8,9^. Public health insurance is designed to cover a specific set of chronic diseases, high-cost conditions, and health issues that are a state priority^10^. In Peru, the population's need for public health insurance is more evident due to the inequities inherent to the existing socioeconomic context, which is accompanied by a deficient health system, even though 97.8% of citizens have some form of health insurance^11^; and there are different types of insurance, such as private insurance and insurance derived from public entities such as the National Police and the Army (2%)^11^.

The Seguro Integral de Salud (SIS) is the most widely used public insurance system and covers more than 71% of the Peruvian population. It provides coverage to people who do not have any other type of insurance as well as to the general population and guarantees access to health services^12,13^. In addition, the SIS, through the Intangible Solidarity Health Fund (FISSAL), finances high-cost benefits. On the other hand, the main Peruvian private insurances are the Private Healthcare Entities (EPS), which are responsible for more than 3% of the total health services provided in the country^11^.

The pandemic affected public health systems worldwide and reduced their capacity to provide healthcare^14^. However, this impact was higher in low- and middle-income countries such as Peru^15^. At the beginning of the pandemic, a significant decrease in the Peruvian health system’s capacity to provide healthcare was observed^16^. Since it is a fragmented system, we cannot measure its capacity accurately or determine whether its healthcare capacity has been recovered, especially for individuals with chronic diseases. For this reason, our study aims to assess the impact of the COVID-19 pandemic on the services provided to individuals with chronic diseases in Peru from 2016 to 2022. Our approach focuses on analyzing the progression of the healthcare services provided by the Peruvian SIS over those years. Healthcare services are represented by the number of single users who receive care every month, which will provide a comprehensive perspective on the progression of the health system and its capacity to provide healthcare to the insured population.

## Method

### Design

We conducted a secondary database analysis according to the data registered by the SIS and FISSAL (insurer that finances the high-cost benefits of the SIS), both belonging to the Peruvian Ministry of Health (MINSA), as well as private Healthcare Entities (EPS) during the 2016–2022 period using an interrupted time series analysis (ITSA).

### Participants

The sample unit consisted of the number of monthly users that received care. Users’ healthcare services were funded by SIS, private EPS, and the FISSAL. All users attending a medical consultation from January 2016 to December 2022 were included. Records with no ID data (national identity document) and with diagnostic inconsistencies (inconsistent diagnosis with sex or age group) were excluded, and only patients 0–99 years old were included.

### Variables

#### Number of users who received care per month

It was defined as the number of users who received care during a given month, such care being funded by SIS, EPS, or FISSAL.

#### Sociodemographic variables

Subjects included were classified by sex (male or female) and age, which included early childhood (0–11 years), adolescence (12–17 years), adulthood (18–64 years), and old age (65 years or over). Additionally, users were grouped by the geographical code of their residence (department, province, and district) and by the district wealth quintile in Peru, based on the data provided by the National Institute of Statistics and Computing of Peru. Moreover, subjects included were grouped based on their healthcare system (SIS, EPS, or FISSAL).

#### Diagnosis of chronic diseases

Diagnoses were classified according to International Classification of Diseases-10 and grouped into 17 diseases based on Mary Charlson’s classification (Myocardial infarction, Congestive heart failure, Peripheral vascular disease, Cerebrovascular disease, Dementia, Chronic pulmonary disease, Connective tissue disease, Ulcer disease, Liver disease, Diabetes or diabetes with organic lesion, Hemiplegia, Kidney disease, Neoplasms, Leukemia, Malignant lymphoma, Solid metastasis, and AIDS)^17^. We defined a case of chronic disease if the participant received a definitive diagnosis of one of Mary Charlson's classifications of chronic disease during the analysis period (2016–2022). We believe this is a valid measure, as these are chronic conditions that, by their nature, do not go away over time.

### Analysis 

We used ITSA, a quasi-experimental approach, to estimate the impact of the COVID-19 pandemic on the services provided by the Peruvian health system. The choice of an ITSA was due to its ability to assess the impact of an event (in this case, the COVID-19 pandemic) over time. Therefore, time series analysis was more appropriate for our data because we used discrete measures that collapsed the number of cases into one month. In addition, ITSA allow us to decompose temporal effects into level and trend changes, providing a richer understanding of how the pandemic changed the use of health services. Furthermore, the use of ITSA has been useful in assessing the impact of the pandemic in different health contexts in Peru^16,18^.

For our study, we established a discrete number of measurements per month. A descriptive analysis was conducted, which included the number of users receiving care per month and their sociodemographic variables. In addition, an ITSA was conducted, considering a censoring period when the Peruvian government declared a state of emergency due to COVID-19 (March 16, 2020), because during this month a strict lockdown was decreed, which reduced the access of public health. A segmented regression analysis with Newey–West standard errors was used for data modeling^19^, and monthly time units were considered. We considered before the pandemic (01 January 2016–28 February 2020) and during the lockdown (01 April 2020–31 December 2022). The estimated impact of the COVID-19 pandemic on outcomes was assessed in terms of the change in the level (intercept) and the change in the slope of the number of users in treatment per month over the time series before and after the interruption of the intervention. The change in the intercept is only the immediate change in the level of prevalence of the outcome, whereas the change in the slope of the number of users receiving care per month reflects the change in the trend of the number of users over time in each quarter. Subgroup analyses were performed for age group, sex, wealth quintile and health subsystem. Values with a 95% hypothesis test (p < 0.05) were considered significant. Users with missing data were excluded. Cumby–Huizinga tests were used with the “actest” command to check autocorrelation^20^. Analyses were conducted with Stata® software, version 17.

### Procedure

Firstly, information from the databases of SIS, FISSAL, and EPS was downloaded through the accessible data platform SUSALUD (http://datos.susalud.gob.pe/). Secondly, the databases were pooled. Thirdly, a data consistency analysis was conducted, which implied confirming that subjects includer’s diagnoses were consistent with their sex or that their age was between 0 and 99 years. Fourthly, the data analyses were conducted, and the research report was written.

### Ethical aspects

Our study uses information from the open data provided by SUSALUD, which is of public access and can be accessed through this link: 10.6084/m9.figshare.25207196. Available data is coded to remain anonymous, and no ethical risks can be identified for the subjects included.

## Results

### Sociodemographic characteristics

We initially analyzed 21,283,302 records, excluding 1922 records because the diagnosis did not match the biological sex of the subjects included (e.g. men with uterine cancer) and 252 records because the diagnosis did not match the age of the subjects included (e.g. children under three years of age diagnosed with pre-eclampsia). No duplicate records were identified. After this review of inconsistencies, less than 0.01% of records were excluded from the analyses.

Our study identified 21,281,128 single users who received care from January 2016 to December 2022 (84 months). Out of 95.5% of the users who received care per month were affiliated with the SIS (n = 20,313,146).

Regarding sociodemographic characteristics, most single users were women (n = 11,517,384; 54.1%), belonged to the lowest wealth quintile (n = 77,021,243; 32.7%) and were of working age, i.e. from 18 to 65 years (n = 5,539,129; 26.0%). As of Charlson’s diseases prevalence, 2.3% of users were diagnosed with diabetes without complications (n = 5,336,813), 1.3% were diagnosed with cancer (n = 3,089,318), 1.3% were diagnosed with chronic pulmonary disease (n = 3,147,185), and a lower percentage was diagnosed with other diseases that are shown in Table 1. Additionally, Table 1 shows the sociodemographic characteristics of users who received care per month before and during the pandemic for each type of Health Insurance Funds Administrative Institutions (IAFAS): SIS, EPS, and FISSAL.

**Table 1:** Sociodemographic characteristics of single users per month, pooled before and during the pandemic (2016–2022).

		All (n = 21,281,128)		SIS (n = 20,313,146)		EPS (n = 1,041,537)		FISSAL (n = 326,098)	
		n	%	n	%	n	%	n	%
Sex	Female	11,517,384	54.1%	11,039,051	54.3%	512,648	49.2%	201,132	61.7%
	Male	9,763,744	45.9%	9,274,095	45.7%	528,889	50.8%	124,966	38.3%
Age group	0–11	6,613,849	31.1%	6,414,147	31.6%	217,274	20.9%	24,757	7.6%
	12–17	2,122,957	10.0%	2,058,859	10.1%	69,552	6.7%	8727	2.7%
	18–64	10,965,666	51.5%	10,296,584	50.7%	719,160	69.0%	186,311	57.1%
	65 or more	1,578,656	7.4%	1,543,556	7.6%	35,551	3.4%	106,303	32.6%
Wealth quartile	Q1	5,539,129	26.0%	5,531,139	27.2%	10,452	1.0%	35,936	11.0%
	Q2	4,548,038	21.4%	4,502,281	22.2%	53,460	5.1%	54,683	16.8%
	Q3	3,804,676	17.9%	3,659,220	18.0%	162,816	15.6%	78,266	24.0%
	Q4	3,688,104	17.3%	3,495,267	17.2%	210,867	20.2%	82,616	25.3%
	Q5	3,701,181	17.4%	3,125,239	15.4%	603,942	58.0%	74,597	22.9%
Pandemic*	Before	16,869,327	79.3%	16,011,798	78.8%	923,116	88.6%	278,757	85.5%
	During	15,750,626	74.0%	15,002,322	73.9%	817,714	78.5%	265,627	81.5%
Health system*	SIS	20,313,146	95.5%	20,313,146	100.0%	75,836	7.3%	323,812	99.3%
	EPS	1,041,537	4.9%	75,836	0.4%	1,041,537	100.0%	999	0.3%
	FISSAL	326,098	1.5%	323,812	1.6%	999	0.1%	326,098	100.0%
Charlson’s classification*	Others	21,247,086	99.8%	20,282,496	99.8%	1,040,421	99.9%	321,940	98.7%
	Cancer	624,948	2.9%	610,016	3.0%	14,539	1.4%	158,425	48.6%
	Metastatic carcinoma	56,104	0.3%	54,912	0.3%	1365	0.1%	27,624	8.5%
	Dementia	37,717	0.2%	35,804	0.2%	2029	0.2%	4956	1.5%
	Diabetes with complications	211,689	1.0%	200,105	1.0%	12,449	1.2%	56,719	17.4%
	Diabetes without complications	1,402,344	6.6%	1,333,632	6.6%	74,735	7.2%	134,405	41.2%
	Cerebrovascular disease	187,407	0.9%	167,681	0.8%	20,761	2.0%	24,326	7.5%
	Mild liver disease	327,339	1.5%	297,491	1.5%	31,589	3.0%	38,527	11.8%
	Moderate or severe liver disease	39,612	0.2%	38,605	0.2%	1118	0.1%	6172	1.9%
	Chronic pulmonary disease	1,373,631	6.5%	1,195,715	5.9%	190,080	18.2%	60,875	18.7%
	Kidney disease	188,812	0.9%	181,427	0.9%	7854	0.8%	80,438	24.7%
	Rheumatic and connective tissue diseases	330,849	1.6%	311,557	1.5%	20,590	2.0%	28,386	8.7%
	Peptic ulcer disease	120,284	0.6%	108,878	0.5%	12,131	1.2%	11,001	3.4%
	Peripheral vascular disease	25,383	0.1%	22,315	0.1%	3183	0.3%	5291	1.6%
	Myocardial infarction	37,417	0.2%	35,246	0.2%	2391	0.2%	6098	1.9%
	Congestive heart failure	173,053	0.8%	162,330	0.8%	11,307	1.1%	33,459	10.3%
	Paraplegia and hemiplegia	40,388	0.2%	38,635	0.2%	1916	0.2%	6348	1.9%
	HIV AIDS	157,771	0.7%	156,896	0.8%	1560	0.1%	7705	2.4%

*SIS* comprehensive health insurance (in Spanish), *EPS* private healthcare entities (in Spanish), *FISSAL* intangible solidarity health fund (in Spanish).

*Values do not add up to 100%.

### Users who received care per month

**Figure 1:**
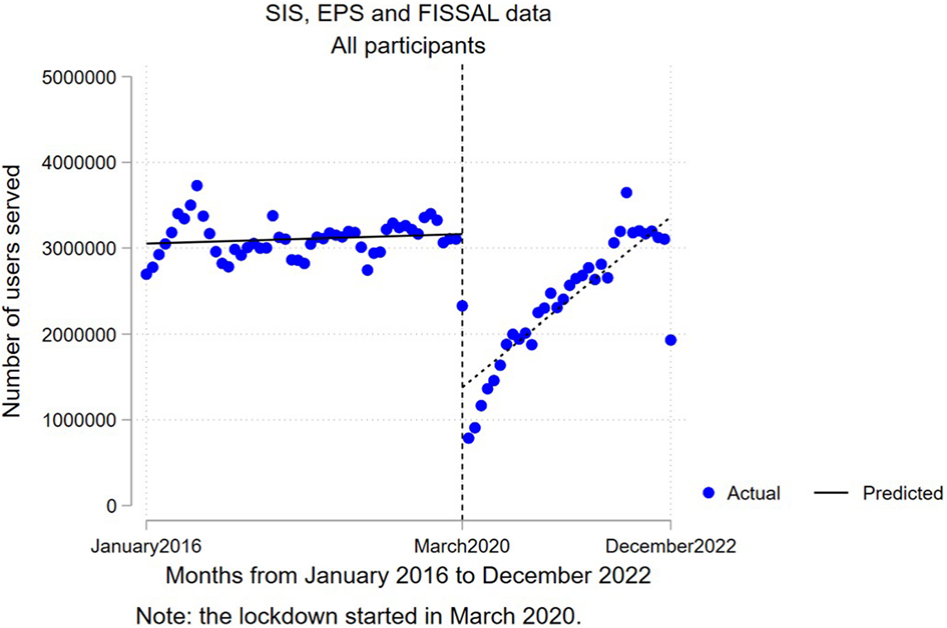
Interrupted time series analysis of the number of users who received care per month from January 2016 to December 2022. *SIS* comprehensive health insurance (in Spanish), *EPS* private healthcare entities (in Spanish), *FISSAL* intangible solidarity health fund (in Spanish).

The pooled analysis of the three types of IAFAS (SIS, EPS, and FISSAL) identified an average decrease of 1,782,446 (95% CI − 2,165,401–− 1,399,490) in the number of users who received care during the first month at the beginning of the pandemic (March 2020) as opposed to the expected values for that month based on the measurements taken before the pandemic (p < 0.001). In addition, during the pandemic months, an average increase of 57,911 (95% CI 35,383–80,439) in the number of new additional single users who received care per month was observed as opposed to the prior month (p < 0.001). Figure 1 shows the change in the number of users for the pooled analyses. In addition, similar results were observed for the age, sex, and wealth quintile groups in the pooled analyses for SIS, EPS, and FISSAL (see Table 2).

**Table 2:** Interrupted time series regression analysis for the number of users who received care.

				Coefficient	*p*	95% CI	
SIS, EPS, and FISSAL	All users		Change in the number of users	− 1,782,446	0.000	− 2,165,401	− 1,399,490
	(See Fig. 1)		Change in trend	57,911	0.000	35,383	80,439
	Sex	Female	Change in the number of users	− 1,070,674	0.000	− 1,304,861	− 836,486
			Change in trend	34,694	0.000	20,983	48,404
		Male	Change in the number of users	− 711,772	0.000	− 861,671	− 561,872
			Change in trend	23,218	0.000	14,341	32,095
	Age group	0 − 11	Change in the number of users	− 775,180	0.000	− 924,827	− 625,532
			Change in trend	22,246	0.000	14,019	30,473
		12 − 17	Change in the number of users	− 194,264	0.000	− 240,556	− 147,971
			Change in trend	6441	0.000	3969	8913
		18 − 64	Change in the number of users	− 655,394	0.000	− 831,290	− 479,498
			Change in trend	26,088	0.000	15,265	36,911
		65 or over	Change in the number of users	− 157,608	0.000	− 190,360	− 124,855
			Change in trend	3136	0.002	1161	5111
	Wealth quintile	Q1	Change in the number of users	− 511,590	0.000	− 632,458	− 390,722
			Change in trend	13,694	0.000	6805	20,582
		Q2	Change in the number of users	− 377,054	0.000	− 462,041	− 292,066
			Change in trend	12,202	0.000	7237	17,167
		Q3	Change in the number of users	− 306,688	0.000	− 370,878	− 242,498
			Change in trend	10,642	0.000	6841	14,442
		Q4	Change in the number of users	− 298,300	0.000	− 355,946	− 240,654
			Change in trend	10,116	0.000	6639	13,594
		Q5	Change in the number of users	− 288,814	0.000	− 348,169	− 229,458
			Change in trend	11,257	0.000	7663	14,851
SIS	All users		Change in the number of users	− 1,667,830	0.000	− 2,028,320	− 1,307,340
	(See Fig. 2C)		Change in trend	51,779	0.000	30,204	73,355
	Sex	Female	Change in the number of users	− 1,018,928	0.000	− 1,242,834	− 795,022
			Change in trend	31,765	0.000	18,513	45,017
		Male	Change in the number of users	− 648,902	0.000	− 787,035	− 510,768
			Change in trend	20,014	0.000	11,614	28,415
	Age group	0 − 11	Change in the number of users	− 740,199	0.000	− 885,050	− 595,348
			Change in trend	20,693	0.000	12,665	28,721
		12 − 17	Change in the number of users	− 186,031	0.000	− 231,256	− 140,806
			Change in trend	6069	0.000	3652	8486
		18 − 64	Change in the number of users	− 590,469	0.000	− 750,171	− 430,767
			Change in trend	22,378	0.000	12,198	32,557
		65 or over	Change in the number of users	− 151,131	0.000	− 182,242	− 120,020
			Change in trend	2640	0.006	766	4514
	Wealth quintile	Q1	Change in the number of users	− 509,975	0.000	− 630,391	− 389,560
			Change in trend	13,543	0.000	6681	20,406
		Q2	Change in the number of users	− 371,488	0.000	− 455,166	− 287,810
			Change in trend	11,797	0.000	6898	16,695
		Q3	Change in the number of users	− 291,621	0.000	− 352,660	− 230,581
			Change in trend	9627	0.000	5970	13,284
		Q4	Change in the number of users	− 277,509	0.000	− 331,114	− 223,903
			Change in trend	8837	0.000	5537	12,136
		Q5	Change in the number of users	− 217,237	0.000	− 263,814	− 170,660
			Change in trend	7976	0.000	4901	11,050
FISSAL	All users		Change in the number of users	− 8634	0.013	− 15,389	− 1878
	(see Fig. 2A)		Change in trend	1128	0.000	748	1509
	Sex	Female	Change in the number of users	− 5163	0.023	− 9593	− 733
			Change in trend	780	0.000	529	1031
		Male	Change in the number of users	− 3470	0.004	− 5823	− 1118
			Change in trend	349	0.000	219	479
	Age group	0 − 11	Change in the number of users	− 956	0.002	− 1538	− 373
			Change in trend	77	0.000	48	106
		12 − 17	Change in the number of users	− 241	0.033	− 462	− 20
			Change in trend	37	0.000	25	49
		18 − 64	Change in the number of users	− 3971	0.057	− 8060	118
			Change in trend	692	0.000	461	923
		65 or over	Change in the number of users	− 3466	0.001	− 5442	− 1490
			Change in trend	323	0.000	212	433
	Wealth quintile	Q1	Change in the number of users	− 930	0.002	− 1502	− 358
			Change in trend	106	0.000	74	139
		Q2	Change in the number of users	− 1517	0.007	− 2612	− 423
			Change in trend	177	0.000	114	239
		Q3	Change in the number of users	− 2010	0.026	− 3775	− 244
			Change in trend	278	0.000	182	375
		Q4	Change in the number of users	− 2169	0.015	− 3898	− 441
			Change in trend	298	0.000	201	394
		Q5	Change in the number of users	− 2007	0.016	− 3631	− 383
			Change in trend	269	0.000	176	363
EPS	All users		Change in the number of users	− 105,982	0.000	− 130,257	− 81,707
	(see Fig. 2B)		Change in trend	5,004	0.000	3929	6078
	Sex	Female	Change in the number of users	− 46,583	0.000	− 56,635	− 36,531
			Change in trend	2149	0.000	1704	2,593
		Male	Change in the number of users	− 59,400	0.000	− 73,715	− 45,084
			Change in trend	2855	0.000	2221	3489
	Age group	0 − 11	Change in the number of users	− 34,025	0.000	− 39,484	− 28,567
			Change in trend	1476	0.000	1232	1720
		12 − 17	Change in the number of users	− 7992	0.000	− 9618	− 6366
			Change in trend	335	0.000	256	414
		18 − 64	Change in the number of users	− 60,954	0.000	− 79,372	− 42,536
			Change in trend	3018	0.000	2201	3836
		65 or over	Change in the number of users	− 3011	0.000	− 4154	− 1868
			Change in trend	174,174	0.000	128	220
	Wealth quintile	Q1	Change in the number of users	− 685	0.000	− 825	− 545
			Change in trend	44	0.000	37	51
		Q2	Change in the number of users	− 4048	0.000	− 4981	− 3116
			Change in trend	228	0.000	185	271
		Q3	Change in the number of users	− 13,058	0.000	− 16,115	− 10,000
			Change in trend	737	0.000	599	875
		Q4	Change in the number of users	− 18,621	0.000	− 23,120	− 14,123
			Change in trend	982	0.000	782	1182
		Q5	Change in the number of users	− 69,570	0.000	− 85,354	− 53,786
			Change in trend	3012	0.000	2317	3707

Change in the number of users at the beginning of the COVID-19 pandemic (change in the intercept). Change in the monthly trend regarding the number of users who received care during the pandemic (change in the slope [interaction]). *SIS* comprehensive health insurance (in Spanish). *EPS* private healthcare entities (in Spanish), *FISSAL* intangible solidarity health fund (in Spanish).

A counterfactual analysis was conducted to determine the impact of the pandemic on the number of single users who received care per month in the three IAFAS types (SIS, EPS, and FISSAL) and reported that the number of users who received care during the pandemic decreased by 26,332,506 (95% CI − 35,205,715–− 17,459,297; p < 0.001). This figure represents the number of users who did not receive medical care from March 2020 to December 2022 because of the pandemic, as opposed to a non-pandemic scenario.

**Figure 2:**
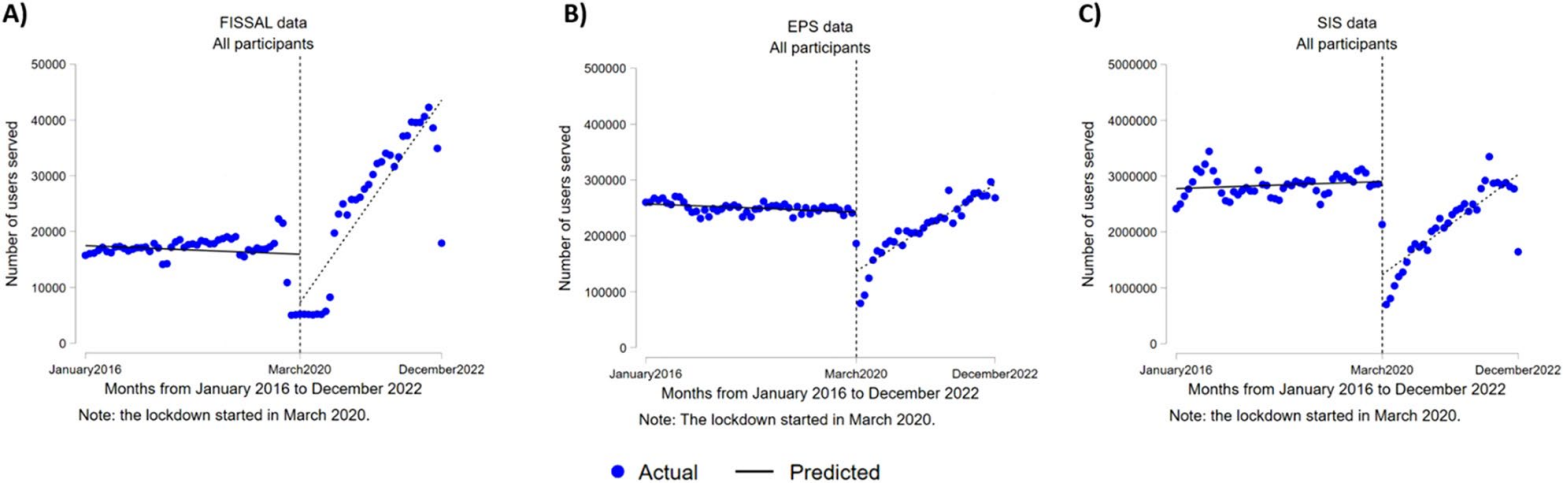
Interrupted time series analysis of the number of users who received care by type of health insurance funds administrative institution. (**A**) For the intangible solidarity health fund (FISSAL). (**B**) For healthcare entities (EPS). (**C**) For the comprehensive health system (SIS).

The individual analyses conducted for each insurance institution indicated similar findings. Regarding users affiliated with the SIS, an average decrease of 2,028,320 in the number of users was observed (95% CI − 2,028,320–− 1,307,340) as opposed to the values expected for that month at the beginning of the COVID-19 pandemic in Peru (see Fig. 2C). Moreover, an average decrease of 8634 was identified for FISSAL (95% CI − 15,389–− 1878) and 105,982 for EPS (95% CI − 130,257–− 81,707) for the number of single users who received care during the first month of the pandemic (see Fig. 2A and B, respectively). This decrease was significant in all cases (p < 0.05).

Regarding the SIS, an increasing trend of 5,004 was observed (95% CI 3929–6078) in the number of single users per month, meaning an average of more than five thousand new single users was observed in the SIS. As of FISSAL and EPS, a trend of 1128 (95% CI 748 to 1509) and 5004 (95% CI 3929 to 6078) was found for new single users per month. The increase was significant in all cases (p < 0.05). Other coefficients in the time series analyses can be seen in Supplementary Material 1.

### Users by type of chronic disease

According to the time series analyses of the users who received care by the SIS, EPS, and FISSAL per month conducted for each group of chronic diseases based on Charlson’s classification^17^, there was a significant decrease in the number of users at the beginning of the pandemic for all diagnostic groups (see Table 3). A considerable decrease was observed in the first month of the pandemic compared to the previous months (p < 0.05). The most significant decreases include individuals with diabetes without complications and chronic pulmonary disease, with a decrease of 33,447 (p < 0.001; 95% CI − 43,781–− 23,114) and 31,365 (p < 0.001; 95% CI − 38,386–− 24,344) users per month, respectively.

**Table 3:** Interrupted time series regression analysis for the number of users who received care by SIS, EPS, and FISSAL by type of Charlson’s disease.

		Coefficient	*p*	95% CI	
Cancer	Change in the number of users	− 21,249	0.000	− 29,070	− 13,428
	Change in trend	1175	0.000	703	1647
Solid metastatic tumor	Change in the number of users	− 716	0.000	− 977	− 455
	Change in trend	67	0.000	44	89
Dementia	Change in the number of users	− 1067	0.000	− 1255	− 879
	Change in trend	22	0.002	8	35
Diabetes with complications	Change in the number of users	− 6204	0.000	− 7336	− 5073
	Change in trend	35	0.347	− 39	109
Diabetes without complications	Change in the number of users	− 33,447	0.000	− 43,781	− 23,114
	Change in trend	1153	0.003	412	1895
Cerebrovascular disease	Change in the number of users	− 3950	0.000	− 4719	− 3182
	Change in trend	77	0.004	25	130
Mild liver disease	Change in the number of users	− 7347	0.000	− 8551	− 6143
	Change in trend	141	0.000	65	218
Moderate liver disease	Change in the number of users	− 649	0.000	− 754	− 544
	Change in trend	12	0.000	6	19
Chronic pulmonary disease	Change in the number of users	− 31,365	0.000	− 38,386	− 24,344
	Change in trend	695	0.000	357	1033
Kidney disease	Change in the number of users	− 8741	0.000	− 10,545	− 6937
	Change in trend	124	0.018	22	227
Rheumatic disease	Change in the number of users	− 7518	0.000	− 9005	− 6030
	Change in trend	185	0.000	87	283
Peptic ulcer	Change in the number of users	− 1206	0.000	− 1453	− 960
	Change in trend	41	0.000	26	57
Peripheral vascular disease	Change in the number of users	− 512	0.000	− 597	− 426
	Change in trend	11	0.000	5	17
Myocardial infarction	Change in the number of users	− 364	0.000	− 462	− 267
	Change in trend	8	0.019	1	14
Congestive heart failure	Change in the number of users	− 3734	0.000	− 4340	− 3129
	Change in trend	46	0.025	6	86
Paraplegia and hemiplegia	Change in the number of users	− 1248	0.000	− 1465	− 1032
	Change in trend	34	0.000	20	49
AIDS/HIV	Change in the number of users	− 5363	0.000	− 6581	− 4144
	Change in trend	101	0.016	19	182

Change in the number of users at the beginning of the COVID-19 pandemic (change in the intercept). Change in the monthly trend regarding the number of users who received care during the pandemic (change in the slope [interaction]). *SIS* comprehensive health insurance (in Spanish). *EPS* private healthcare entities (in Spanish). *FISSAL* intangible solidarity health fund (in Spanish).

However, during the pandemic, we observed an increasing number of users with these diseases in all Charlson’s diagnosis groups (p < 0.05), except for monthly users with diabetes with complications. A significant increase was found in the number of monthly users from the groups of cancer and diabetes without complications, with 1,175 (p < 0.001; 95% CI 703–1647) and 1153 (p = 0.003; 95% CI 412–1895) new users per month, respectively. Table 3 shows the rest of Charlson’s diseases, and Supplementary Material 2 shows other indicators of the time series analyses.

## Discussion

Our study evaluated the impact of the COVID-19 pandemic on services provided to people with chronic diseases in Peru from 2016 to 2022 and showed a significant decrease in the number of users who received care during the first month of the pandemic as opposed to prior measurements. This decrease was observed in the pooled analysis of IAFAS (SIS, EPS, and FISSAL) and the individual analyses conducted for each type of insurer. In addition, we identified an average monthly increase in the number of users who received care during the pandemic as opposed to the previous month, which suggests a gradual recovery in the demand for healthcare services.

Our findings revealed a similar pattern regarding the number of users who received care per month and had chronic diseases based on Charlson’s classification^17^. There was a significant decrease in the number of users at the beginning of the pandemic, followed by an increasing trend during the pandemic, except for individuals with diabetes and complications. Nevertheless, we should highlight that the proportion of individuals with Charlson’s chronic diseases was lower in all cases during the pandemic than before. This finding suggests that many subjects with chronic diseases could not receive the care they needed during the pandemic since the health system prioritized responding to and caring for COVID-19 cases, failing to meet other demands^21^.

Our study identified that the Peruvian health system achieved a partial or complete recovery and could provide care for several patients similar to the one before the pandemic. However, there is still an open gap in the care provided during the pandemic. From March 2020 to December 2022, 26,332,506 individuals failed to receive care, which shows a significant interruption in healthcare and highlights the negative consequences of the pandemic regarding access to healthcare services. This occurred despite the implementation of strategies such as telemedicine to expand the services offered (teleinterconsultation, teleconsultation, teleorientation, telemonitoring, and diagnosis telesupport) as well as policies addressing the four axes of digital health (telemedicine, teletraining, telemanagement and teleinformation, and education and communication)^22^. We should highlight that users who did not receive care may have experienced considerable delay in the diagnosis, treatment, and follow-up of their conditions, which could potentially affect their long-term health and well-being. In addition, closing this gap would require significant public and private investments, which is discouraging given the finite healthcare capacity of the system. Even though the budget for health insurance significantly increased in Peru during the pandemic to address this health emergency^23^, its negative impact on healthcare access could not be avoided. Complete recovery of the healthcare capacity lost during the pandemic will require consistent efforts and effective strategies to address this cumulative delay and ensure equitable access to healthcare for all affected users.

During the first year of the pandemic, a systematic review encompassing 20 countries reported a 37% reduction in the general use of health services; this value is higher in subjects with chronic or severe health conditions^14^. In addition, a systematic review of 22 studies conducted in low- and middle-income African countries suggested that the decrease in the healthcare services provided during the pandemic may be due to the lack of adequate equipment, protocols to face health crises, and solid policies for remote care and limited resources^24^. Similar situations have been observed in Peru since its fragmented health system, lack of specialized health personnel before the pandemic and death of health personnel or leaves granted to them during the pandemic, as well as limited health budget, are the reasons why adequate care could not be provided^25^. This may account for the considerably reduced number of users who received care at the beginning of the pandemic. We should highlight that a limited number of health professionals available in the Peruvian health system is still being estimated. Medical training and residencies were delayed during the pandemic, so the chance to recover healthcare capacity may be obstructed by issues associated with the availability of human resources, as well as the fact that training opportunities are not equitably distributed throughout regions^26^.

Even though there still is an open gap in the number of healthcare services provided by the overall Peruvian health system during the pandemic up to 2022, this gap has been closed in some areas. An analysis of the mental care provided in Peru presented that, after 9 months, community mental health centers could provide care to the same number of users that had received care before the pandemic^16^. This achievement may be partially due to the increase in the prevalence of mental health issues during the pandemic in Peru^18^, resulting in a higher demand. Additionally, it is important to consider that this successful recovery may be due to the implementation of specific strategies during the COVID-19 pandemic and the increased resources destined for mental health^27^.

We consider two possible explanations for the fact that the number of people with diabetes with complications did not increase significantly during the pandemic. First, the nature of having diabetes with complications may have meant that patients with an additional complication, such as diabetic foot, were unable to access necessary care during the pandemic, resulting in deaths due to lack of access to timely care^28^. Second, having diabetes increased the likelihood of complications and mortality in the early years of the pandemic, as COVID-19 infection in these patients had a more severe prognosis compared to individuals without chronic disease^29^.

Some results were not equivalent between the pooled analysis and each of the Peruvian health subsystems, as in the case of FISSAL with specific individuals aged 18 to 64 years. This may be due to differences in the specific populations served by each system, for example, FISSAL covers care for high-cost diseases, which could generate different conditions than the other health subsystems in the context of the pandemic.

### Strengths and limitations

The main strength of our study is that it contains the highest amount of information on the Peruvian health system collected up to date to assess the pandemic’s impact on healthcare services. As a result, we have acquired a wide and representative perspective of the situation and gained a better understanding of the implications of the pandemic on the access to and use of healthcare services in Peru. Given the considerable sample size of the study, our findings are more robust and can be generalized.

For the other hand, there are some limitations. Firstly, even though a significant percentage of all healthcare services of the Peruvian health system were included, other important subsystems were excluded, such as the Social Security Administration of Peru (EsSalud), which finances the services provided to around 29.9% of the Peruvian population. This may imply that our findings only partially represent some aspects of the medical care provided during the pandemic. Secondly, Peru does not have open-access clinical databases that comply with international health standards. Despite that, our study uses an administrative service database that includes the leading national health financing entities. Third, we use data from different subsystems of the Peruvian health system, which are subject to standardization processes for integration into a single database. Although there may be some inconsistent or erroneous cases in the records, we expect the proportion of these data to be minimal. In addition, these limitations are common in large health databases in low- and middle-income countries. Fourth, the variable of chronic disease diagnoses in our database may suffer from underreporting, recording errors, or other problems typical of low- and middle-income countries. However, we believe that these errors, if they exist, would be minimal and would not have a significant impact on the results of our study, so the findings should retain their validity.

## Conclusions

Our study aimed to assess the impact of the COVID-19 pandemic on a wide set of users, with a total of 21,281,128 users receiving care per month in Peru from 2016 to 2022. Our findings reported a significant decrease in the number of users who received care during the first month of the pandemic, followed by an increasing trend (March 2020–December 2022). A positive aspect that should be highlighted is that the Peruvian health system recovered its capacity to provide healthcare since the number of users who received care per month by December 2022 was similar to that before the pandemic. We should mention that an estimated gap of 26,332,506 users who failed to receive care per month was observed during the pandemic.

Based on these findings, policies and strategies should be developed to strengthen both the public and private Peruvian health systems. Approaching this healthcare gap is essential to ensure that users who failed to receive care during the pandemic can access the necessary healthcare services. These policies should focus on improving the capacity to provide healthcare and the distribution of resources, as well as applying effective measures to close the healthcare gap and mitigate the long-term negative effects on the health and well-being of the Peruvian population. Additionally, databases that can be operated between subsystems and comply with international health standards should be developed to be used to make health-related decisions.

### Supplementary Information

Supplementary Information.

## Data Availability

This research used the freely accessible databases, available on the open data platform of SUSALUD (http://datos.susalud.gob.pe/dataset). It should be noted that the data are available in an anonymized form and do not allow identification of the users, therefore, the approval of an ethics committee will not be necessary, as we will not be working directly with the subjects included.
