# Supplementary material for: Impact of the COVID-19 pandemic on the services provided by the Peruvian health system: an analysis of people with chronic diseases
Source: Sci Rep. 2024 Feb 13;14:3664. doi: 10.1038/s41598-024-54275-7 (PMC10864310; doi:10.1038/s41598-024-54275-7)
Supplement: Supplementary file 1 — Supplementary Information. [file 41598_2024_54275_MOESM1_ESM.docx]

**Supplementary material 1.** Other coefficients of the Interrupted time series regression analysis for the number of users who received care.

|  |  |  |  | Coefficient | p | 95% CI | |
| --- | --- | --- | --- | --- | --- | --- | --- |
| SIS, EPS, and FISSAL | All users |  | Slope before the intervention | 2214 | 0.463 | -3766 | 8194 |
|  | (See Figure 1) |  | Change in the intercept | -1782446 | 0.000 | -2165401 | -1399490 |
|  |  |  | Change in the slope (interaction) | 57911 | 0,000 | 35383 | 80439 |
|  |  |  | Intersection | 3050985 | 0.000 | 2851024 | 3250945 |
|  |  |  | Linear trend after the intervention | 60125 | 0.000 | 38345.51 | 81904.92 |
|  | Sex | Female | Slope before the intervention | 1264 | 0.430 | -1907 | 4435 |
|  |  |  | Change in the intercept | -1070674 | 0,000 | -1304861 | -836486 |
|  |  |  | Change in the slope (interaction) | 34694 | 0.000 | 20983 | 48404 |
|  |  |  | Intersection | 1884216 | 0.000 | 1779213 | 1989220 |
|  |  |  | Linear trend after the intervention | 35957 | 0.000 | 22598.33 | 49316.65 |
|  |  | Male | Slope before the intervention | 950 | 0.506 | -1877 | 3777 |
|  |  |  | Change in the intercept | -711772 | 0.000 | -861671 | -561872 |
|  |  |  | Change in the slope (interaction) | 23218 | 0,000 | 14341 | 32095 |
|  |  |  | Intersection | 1166768 | 0.000 | 1071301 | 1262235 |
|  |  |  | Linear trend after the intervention | 24168 | 0.000 | 15712.44 | 32623.01 |
|  | Age group | 0-11 | Slope before the intervention | -1021 | 0.591 | -4790 | 2748 |
|  |  |  | Change in the intercept | -775180 | 0.000 | -924827 | -625532 |
|  |  |  | Change in the slope (interaction) | 22246 | 0.000 | 14019 | 30473 |
|  |  |  | Intersection | 1209983 | 0.000 | 1080606 | 1339360 |
|  |  |  | Linear trend after the intervention | 21225 | 0.000 | 13845.98 | 28603.04 |
|  |  | 12-17 | Slope before the intervention | 41 | 0.949 | -1237 | 1318 |
|  |  |  | Change in the intercept | -194264 | 0.000 | -240556 | -147971 |
|  |  |  | Change in the slope (interaction) | 6441 | 0.000 | 3969 | 8913 |
|  |  |  | Intersection | 271283 | 0.000 | 231716 | 310851 |
|  |  |  | Linear trend after the intervention | 6482 | 0.000 | 4312.134 | 8652.153 |
|  |  | 18-64 | Slope before the intervention | 1476 | 0.022 | 217 | 2736 |
|  |  |  | Change in the intercept | -655394 | 0.000 | -831290 | -479498 |
|  |  |  | Change in the slope (interaction) | 26088 | 0.000 | 15265 | 36911 |
|  |  |  | Intersection | 1356660 | 0.000 | 1317043 | 1396276 |
|  |  |  | Linear trend after the intervention | 27564 | 0.000 | 16845.88 | 38282.56 |
|  |  | 65 or over | Slope before the intervention | 1718 | 0.000 | 1484 | 1952 |
|  |  |  | Change in the intercept | -157608 | 0.000 | -190360 | -124855 |
|  |  |  | Change in the slope (interaction) | 3136 | 0.002 | 1161 | 5111 |
|  |  |  | Intersection | 213059 | 0.000 | 206165 | 219953 |
|  |  |  | Linear trend after the intervention | 4854 | 0.000 | 2896.706 | 6811.971 |
|  | Wealth quintile | Q1 | Slope before the intervention | 516 | 0.636 | -1648 | 2680 |
|  |  |  | Change in the intercept | -511590 | 0.000 | -632458 | -390722 |
|  |  |  | Change in the slope (interaction) | 13694 | 0.000 | 6805 | 20582 |
|  |  |  | Intersection | 1011124 | 0.000 | 940560 | 1081688 |
|  |  |  | Linear trend after the intervention | 14210 | 0.000 | 7607.253 | 20812.22 |
|  |  | Q2 | Slope before the intervention | 361 | 0.605 | -1022 | 1745 |
|  |  |  | Change in the intercept | -377054 | 0.000 | -462041 | -292066 |
|  |  |  | Change in the slope (interaction) | 12202 | 0.000 | 7237 | 17167 |
|  |  |  | Intersection | 648218 | 0.000 | 602474 | 693963 |
|  |  |  | Linear trend after the intervention | 12563 | 0.000 | 7778.203 | 17347.84 |
|  |  | Q3 | Slope before the intervention | 519 | 0.279 | -429 | 1467 |
|  |  |  | Change in the intercept | -306688 | 0.000 | -370878 | -242498 |
|  |  |  | Change in the slope (interaction) | 10642 | 0.000 | 6841 | 14442 |
|  |  |  | Intersection | 461599 | 0.000 | 430033 | 493165 |
|  |  |  | Linear trend after the intervention | 11161 | 0.000 | 7491.657 | 14829.69 |
|  |  | Q4 | Slope before the intervention | 633 | 0.157 | -248 | 1513 |
|  |  |  | Change in the intercept | -298300 | 0.000 | -355946 | -240654 |
|  |  |  | Change in the slope (interaction) | 10116 | 0.000 | 6639 | 13594 |
|  |  |  | Intersection | 434027 | 0.000 | 404491 | 463563 |
|  |  |  | Linear trend after the intervention | 10749 | 0.000 | 7398.515 | 14099.64 |
|  |  | Q5 | Slope before the intervention | 185 | 0.649 | -622 | 993 |
|  |  |  | Change in the intercept | -288814 | 0.000 | -348169 | -229458 |
|  |  |  | Change in the slope (interaction) | 11257 | 0.000 | 7663 | 14851 |
|  |  |  | Intersection | 496016 | 0.000 | 468154 | 523879 |
|  |  |  | Linear trend after the intervention | 11443 | 0.000 | 7946.166 | 14939.23 |
| SIS | All users |  | Slope before the intervention | 2534 | 0.392 | -3329 | 8397 |
|  | (See Figure 1) |  | Change in the intercept | -1667830 | 0.000 | -2028320 | -1307340 |
|  |  |  | Change in the slope (interaction) | 51779 | 0.000 | 30204 | 73355 |
|  |  |  | Intersection | 2776316 | 0.000 | 2580155 | 2972477 |
|  |  |  | Linear trend after the intervention | 54313 | 0.000 | 33504.21 | 75122.46 |
|  | Sex | Female | Slope before the intervention | 1449 | 0.356 | -1658 | 4556 |
|  |  |  | Change in the intercept | -1018928 | 0.000 | -1242834 | -795022 |
|  |  |  | Change in the slope (interaction) | 31765 | 0.000 | 18513 | 45017 |
|  |  |  | Intersection | 1758552 | 0.000 | 1655573 | 1861532 |
|  |  |  | Linear trend after the intervention | 33214 | 0.000 | 20320.63 | 46107.68 |
|  |  | Male | Slope before the intervention | 1085 | 0.439 | -1691 | 3861 |
|  |  |  | Change in the intercept | -648902 | 0.000 | -787035 | -510768 |
|  |  |  | Change in the slope (interaction) | 20014 | 0.000 | 11614 | 28415 |
|  |  |  | Intersection | 1017764 | 0.000 | 924024 | 1111504 |
|  |  |  | Linear trend after the intervention | 21099 | 0.000 | 13135.88 | 29062.48 |
|  | Age group | 0-11 | Slope before the intervention | -817 | 0.662 | -4527 | 2892 |
|  |  |  | Change in the intercept | -740199 | 0.000 | -885050 | -595348 |
|  |  |  | Change in the slope (interaction) | 20693 | 0.000 | 12665 | 28721 |
|  |  |  | Intersection | 1149465 | 0.000 | 1022027 | 1276903 |
|  |  |  | Linear trend after the intervention | 19875 | 0.000 | 12695.23 | 27055.51 |
|  |  | 12-17 | Slope before the intervention | 11 | 0.986 | -1261 | 1283 |
|  |  |  | Change in the intercept | -186031 | 0.000 | -231256 | -140806 |
|  |  |  | Change in the slope (interaction) | 6069 | 0.000 | 3652 | 8486 |
|  |  |  | Intersection | 257963 | 0.000 | 218538 | 297388 |
|  |  |  | Linear trend after the intervention | 6080 | 0.000 | 3971.365 | 8189.036 |
|  |  | 18-64 | Slope before the intervention | 1667 | 0.007 | 460 | 2874 |
|  |  |  | Change in the intercept | -590469 | 0.000 | -750171 | -430767 |
|  |  |  | Change in the slope (interaction) | 22378 | 0.000 | 12198 | 32557 |
|  |  |  | Intersection | 1172773 | 0.000 | 1135222 | 1210325 |
|  |  |  | Linear trend after the intervention | 24045 | 0.000 | 13973.85 | 34115.4 |
|  |  | 65 or over | Slope before the intervention | 1673 | 0.000 | 1437 | 1909 |
|  |  |  | Change in the intercept | -151131 | 0.000 | -182242 | -120020 |
|  |  |  | Change in the slope (interaction) | 2640 | 0.006 | 766 | 4514 |
|  |  |  | Intersection | 196114 | 0.000 | 189270 | 202959 |
|  |  |  | Linear trend after the intervention | 4313 | 0.000 | 2461.728 | 6164.546 |
|  | Wealth quintile | Q1 | Slope before the intervention | 535 | 0.624 | -1626 | 2696 |
|  |  |  | Change in the intercept | -509975 | 0.000 | -630391 | -389560 |
|  |  |  | Change in the slope (interaction) | 13543 | 0.000 | 6681 | 20406 |
|  |  |  | Intersection | 1007313 | 0.000 | 936751 | 1077874 |
|  |  |  | Linear trend after the intervention | 14078 | 0.000 | 7503.356 | 20652.96 |
|  |  | Q2 | Slope before the intervention | 396 | 0.570 | -983 | 1775 |
|  |  |  | Change in the intercept | -371488 | 0.000 | -455166 | -287810 |
|  |  |  | Change in the slope (interaction) | 11797 | 0.000 | 6898 | 16695 |
|  |  |  | Intersection | 634984 | 0.000 | 589327 | 680640 |
|  |  |  | Linear trend after the intervention | 12192 | 0.000 | 7476.626 | 16908.34 |
|  |  | Q3 | Slope before the intervention | 585 | 0.217 | -350 | 1521 |
|  |  |  | Change in the intercept | -291621 | 0.000 | -352660 | -230581 |
|  |  |  | Change in the slope (interaction) | 9627 | 0.000 | 5970 | 13284 |
|  |  |  | Intersection | 423869 | 0.000 | 392735 | 455003 |
|  |  |  | Linear trend after the intervention | 10212 | 0.000 | 6690.881 | 13733.12 |
|  |  | Q4 | Slope before the intervention | 686 | 0.115 | -172 | 1543 |
|  |  |  | Change in the intercept | -277509 | 0.000 | -331114 | -223903 |
|  |  |  | Change in the slope (interaction) | 8837 | 0.000 | 5537 | 12136 |
|  |  |  | Intersection | 383371 | 0.000 | 354586 | 412156 |
|  |  |  | Linear trend after the intervention | 9522 | 0.000 | 6352.766 | 12691.37 |
|  |  | Q5 | Slope before the intervention | 333 | 0.368 | -398 | 1063 |
|  |  |  | Change in the intercept | -217237 | 0.000 | -263814 | -170660 |
|  |  |  | Change in the slope (interaction) | 7976 | 0.000 | 4901 | 11050 |
|  |  |  | Intersection | 326780 | 0.000 | 301854 | 351706 |
|  |  |  | Linear trend after the intervention | 8309 | 0.000 | 5333.473 | 11283.77 |
| FISSAL | All users |  | Slope before the intervention | -31 | 0.573 | -138 | 77 |
|  | (See Figure 1) |  | Change in the intercept | -8634 | 0.013 | -15389 | -1878 |
|  |  |  | Change in the slope (interaction) | 1128 | 0.000 | 748 | 1509 |
|  |  |  | Intersection | 17470 | 0.000 | 15688 | 19252 |
|  |  |  | Linear trend after the intervention | 1098 | 0.000 | 734.1756 | 1461.317 |
|  | Sex | Female | Slope before the intervention | -56 | 0.102 | -124 | 11 |
|  |  |  | Change in the intercept | -5163 | 0.023 | -9593 | -733 |
|  |  |  | Change in the slope (interaction) | 780 | 0.000 | 529 | 1031 |
|  |  |  | Intersection | 11980 | 0.000 | 10822 | 13137 |
|  |  |  | Linear trend after the intervention | 723 | 0.000 | 482.7033 | 964.1166 |
|  |  | Male | Slope before the intervention | 26 | 0.216 | -15 | 66 |
|  |  |  | Change in the intercept | -3470 | 0.004 | -5823 | -1118 |
|  |  |  | Change in the slope (interaction) | 349 | 0.000 | 219 | 479 |
|  |  |  | Intersection | 5491 | 0.000 | 4843 | 6138 |
|  |  |  | Linear trend after the intervention | 374 | 0.000 | 251.395 | 497.2782 |
|  | Age group | 0-11 | Slope before the intervention | 11 | 0.064 | -1 | 23 |
|  |  |  | Change in the intercept | -956 | 0.002 | -1538 | -373 |
|  |  |  | Change in the slope (interaction) | 77 | 0.000 | 48 | 106 |
|  |  |  | Intersection | 527 | 0.000 | 345 | 709 |
|  |  |  | Linear trend after the intervention | 88 | 0.000 | 61.77571 | 114.4024 |
|  |  | 12-17 | Slope before the intervention | 0 | 0.777 | -3 | 4 |
|  |  |  | Change in the intercept | -241 | 0.033 | -462 | -20 |
|  |  |  | Change in the slope (interaction) | 37 | 0.000 | 25 | 49 |
|  |  |  | Intersection | 317 | 0.000 | 266 | 368 |
|  |  |  | Linear trend after the intervention | 37 | 0.000 | 25.6507 | 48.97635 |
|  |  | 18-64 | Slope before the intervention | -47 | 0.112 | -104 | 11 |
|  |  |  | Change in the intercept | -3971 | 0.057 | -8060 | 118 |
|  |  |  | Change in the slope (interaction) | 692 | 0.000 | 461 | 923 |
|  |  |  | Intersection | 11481 | 0.000 | 10474 | 12488 |
|  |  |  | Linear trend after the intervention | 645 | 0.000 | 422.4616 | 868.2467 |
|  |  | 65 or over | Slope before the intervention | 4 | 0.812 | -33 | 41 |
|  |  |  | Change in the intercept | -3466 | 0.001 | -5442 | -1490 |
|  |  |  | Change in the slope (interaction) | 323 | 0.000 | 212 | 433 |
|  |  |  | Intersection | 5146 | 0.000 | 4541 | 5750 |
|  |  |  | Linear trend after the intervention | 327 | 0.000 | 222.9874 | 430.9921 |
|  | Wealth quintile | Q1 | Slope before the intervention | -8 | 0.129 | -18 | 2 |
|  |  |  | Change in the intercept | -930 | 0.002 | -1502 | -358 |
|  |  |  | Change in the slope (interaction) | 106 | 0.000 | 74 | 139 |
|  |  |  | Intersection | 1906 | 0.000 | 1724 | 2088 |
|  |  |  | Linear trend after the intervention | 99 | 0.000 | 67.64427 | 129.4219 |
|  |  | Q2 | Slope before the intervention | -5 | 0.577 | -23 | 13 |
|  |  |  | Change in the intercept | -1517 | 0.007 | -2612 | -423 |
|  |  |  | Change in the slope (interaction) | 177 | 0.000 | 114 | 239 |
|  |  |  | Intersection | 3071 | 0.000 | 2752 | 3389 |
|  |  |  | Linear trend after the intervention | 172 | 0.000 | 112.3095 | 230.9595 |
|  |  | Q3 | Slope before the intervention | -1 | 0.931 | -27 | 25 |
|  |  |  | Change in the intercept | -2010 | 0.026 | -3775 | -244 |
|  |  |  | Change in the slope (interaction) | 278 | 0.000 | 182 | 375 |
|  |  |  | Intersection | 4048 | 0.000 | 3606 | 4490 |
|  |  |  | Linear trend after the intervention | 277 | 0.000 | 184.5501 | 369.6567 |
|  |  | Q4 | Slope before the intervention | -6 | 0.648 | -35 | 22 |
|  |  |  | Change in the intercept | -2169 | 0.015 | -3898 | -441 |
|  |  |  | Change in the slope (interaction) | 298 | 0.000 | 201 | 394 |
|  |  |  | Intersection | 4396 | 0.000 | 3946 | 4847 |
|  |  |  | Linear trend after the intervention | 291 | 0.000 | 199.3072 | 382.9932 |
|  |  | Q5 | Slope before the intervention | -10 | 0.441 | -36 | 16 |
|  |  |  | Change in the intercept | -2007 | 0.016 | -3631 | -383 |
|  |  |  | Change in the slope (interaction) | 269 | 0.000 | 176 | 363 |
|  |  |  | Intersection | 4049 | 0.000 | 3630 | 4469 |
|  |  |  | Linear trend after the intervention | 259 | 0.000 | 169.8473 | 348.8033 |
| EPS | All users |  | Slope before the intervention | -289 | 0.003 | -477 | -102 |
|  | (See Figure 1) |  | Change in the intercept | -105982 | 0.000 | -130257 | -81707 |
|  |  |  | Change in the slope (interaction) | 5004 | 0.000 | 3929 | 6078 |
|  |  |  | Intersection | 257198 | 0.000 | 250474 | 263922 |
|  |  |  | Linear trend after the intervention | 4714 | 0.000 | 3653.627 | 5774.644 |
|  | Sex | Female | Slope before the intervention | -129 | 0.007 | -221 | -37 |
|  |  |  | Change in the intercept | -46583 | 0.000 | -56635 | -36531 |
|  |  |  | Change in the slope (interaction) | 2149 | 0.000 | 1704 | 2593 |
|  |  |  | Intersection | 113685 | 0.000 | 110424 | 116945 |
|  |  |  | Linear trend after the intervention | 2020 | 0.000 | 1583.009 | 2456.839 |
|  |  | Male | Slope before the intervention | -160 | 0.002 | -258 | -63 |
|  |  |  | Change in the intercept | -59400 | 0.000 | -73715 | -45084 |
|  |  |  | Change in the slope (interaction) | 2855 | 0.000 | 2221 | 3489 |
|  |  |  | Intersection | 143514 | 0.000 | 140010 | 147017 |
|  |  |  | Linear trend after the intervention | 2694 | 0.000 | 2067.089 | 3321.333 |
|  | Age group | 0-11 | Slope before the intervention | -215 | 0.000 | -303 | -127 |
|  |  |  | Change in the intercept | -34025 | 0.000 | -39484 | -28567 |
|  |  |  | Change in the slope (interaction) | 1476 | 0.000 | 1232 | 1720 |
|  |  |  | Intersection | 59991 | 0.000 | 57087 | 62894 |
|  |  |  | Linear trend after the intervention | 1261 | 0.000 | 1029.963 | 1492.135 |
|  |  | 12-17 | Slope before the intervention | 30 | 0.000 | 14 | 45 |
|  |  |  | Change in the intercept | -7992 | 0.000 | -9618 | -6366 |
|  |  |  | Change in the slope (interaction) | 335 | 0.000 | 256 | 414 |
|  |  |  | Intersection | 13003 | 0.000 | 12591 | 13415 |
|  |  |  | Linear trend after the intervention | 365 | 0.000 | 288.0873 | 441.1711 |
|  |  | 18-64 | Slope before the intervention | -144 | 0.010 | -253 | -35 |
|  |  |  | Change in the intercept | -60954 | 0.000 | -79372 | -42536 |
|  |  |  | Change in the slope (interaction) | 3018 | 0.000 | 2201 | 3836 |
|  |  |  | Intersection | 172406 | 0.000 | 168570 | 176242 |
|  |  |  | Linear trend after the intervention | 2874 | 0.000 | 2062.53 | 3685.961 |
|  |  | 65 or over | Slope before the intervention | 40 | 0.000 | 23 | 58 |
|  |  |  | Change in the intercept | -3011 | 0.000 | -4154 | -1868 |
|  |  |  | Change in the slope (interaction) | 174 | 0.000 | 128 | 220 |
|  |  |  | Intersection | 11799 | 0.000 | 11522 | 12075 |
|  |  |  | Linear trend after the intervention | 214 | 0.000 | 172.0617 | 256.3615 |
|  | Wealth quintile | Q1 | Slope before the intervention | -11 | 0.000 | -14 | -8 |
|  |  |  | Change in the intercept | -685 | 0.000 | -825 | -545 |
|  |  |  | Change in the slope (interaction) | 44 | 0.000 | 37 | 51 |
|  |  |  | Intersection | 1905 | 0.000 | 1826 | 1985 |
|  |  |  | Linear trend after the intervention | 33 | 0.000 | 27.05624 | 39.03666 |
|  |  | Q2 | Slope before the intervention | -29 | 0.000 | -42 | -17 |
|  |  |  | Change in the intercept | -4048 | 0.000 | -4981 | -3116 |
|  |  |  | Change in the slope (interaction) | 228 | 0.000 | 185 | 271 |
|  |  |  | Intersection | 10164 | 0.000 | 9746 | 10581 |
|  |  |  | Linear trend after the intervention | 199 | 0.000 | 158.2689 | 239.5459 |
|  |  | Q3 | Slope before the intervention | -65 | 0.000 | -98 | -33 |
|  |  |  | Change in the intercept | -13058 | 0.000 | -16115 | -10000 |
|  |  |  | Change in the slope (interaction) | 737 | 0.000 | 599 | 875 |
|  |  |  | Intersection | 33682 | 0.000 | 32552 | 34813 |
|  |  |  | Linear trend after the intervention | 672 | 0.000 | 537.9861 | 805.1524 |
|  |  | Q4 | Slope before the intervention | -46 | 0.021 | -86 | -7 |
|  |  |  | Change in the intercept | -18621 | 0.000 | -23120 | -14123 |
|  |  |  | Change in the slope (interaction) | 982 | 0.000 | 782 | 1182 |
|  |  |  | Intersection | 46260 | 0.000 | 44898 | 47622 |
|  |  |  | Linear trend after the intervention | 936 | 0.000 | 739.7866 | 1131.935 |
|  |  | Q5 | Slope before the intervention | -137 | 0.016 | -249 | -26 |
|  |  |  | Change in the intercept | -69570 | 0.000 | -85354 | -53786 |
|  |  |  | Change in the slope (interaction) | 3012 | 0.000 | 2317 | 3707 |
|  |  |  | Intersection | 165187 | 0.000 | 161185 | 169190 |
|  |  |  | Linear trend after the intervention | 2875 | 0.000 | 2185.014 | 3564.489 |

SIS = Comprehensive Health Insurance (in Spanish). EPS= private Healthcare Entities (in Spanish). FISSAL = Intangible Solidarity Health Fund (in Spanish).

**Supplementary material 2.** Other coefficients of the interrupted time series regression analysis for the number of users who received care by SIS, EPS, and FISSAL by type of Charlson’s disease.

|  |  | Coefficient | p | 95% CI | |
| --- | --- | --- | --- | --- | --- |
| Cancer | Slope before the intervention | 110 | 0.001 | 46 | 174 |
|  | Change in the intercept | -21249 | 0.000 | -29070 | -13428 |
|  | Change in the slope (interaction) | 1175 | 0.000 | 703 | 1647 |
|  | Intersection | 32958 | 0.000 | 31482 | 34434 |
|  | Linear trend after the intervention | 1285 | 0.000 | 816.366 | 1753.736 |
| Solid metastatic tumor | Slope before the intervention | 11 | 0.000 | 9 | 13 |
|  | Change in the intercept | -716 | 0.000 | -977 | -455 |
|  | Change in the slope (interaction) | 67 | 0.000 | 44 | 89 |
|  | Intersection | 486 | 0.000 | 431 | 542 |
|  | Linear trend after the intervention | 78 | 0.000 | 55.71229 | 100.6639 |
| Dementia | Slope before the intervention | 16 | 0.000 | 14 | 18 |
|  | Change in the intercept | -1067 | 0.000 | -1255 | -879 |
|  | Change in the slope (interaction) | 22 | 0.002 | 8 | 35 |
|  | Intersection | 666 | 0.000 | 620 | 713 |
|  | Linear trend after the intervention | 38 | 0.000 | 24.59783 | 51.39514 |
| Diabetes with complications | Slope before the intervention | 103 | 0.000 | 93 | 114 |
|  | Change in the intercept | -6204 | 0.000 | -7336 | -5073 |
|  | Change in the slope (interaction) | 35 | 0.347 | -39 | 109 |
|  | Intersection | 4259 | 0.000 | 3898 | 4620 |
|  | Linear trend after the intervention | 138 | 0.000 | 65.54033 | 211.3639 |
| Diabetes without complications | Slope before the intervention | 726 | 0.000 | 618 | 834 |
|  | Change in the intercept | -33447 | 0.000 | -43781 | -23114 |
|  | Change in the slope (interaction) | 1153 | 0.003 | 412 | 1895 |
|  | Intersection | 39240 | 0.000 | 36096 | 42383 |
|  | Linear trend after the intervention | 1879 | 0.000 | 1147.499 | 2610.734 |
| Cerebrovascular disease | Slope before the intervention | 39 | 0.000 | 33 | 45 |
|  | Change in the intercept | -3950 | 0.000 | -4719 | -3182 |
|  | Change in the slope (interaction) | 77 | 0.004 | 25 | 130 |
|  | Intersection | 3851 | 0.000 | 3657 | 4044 |
|  | Linear trend after the intervention | 116 | 0.000 | 64.57138 | 168.2271 |
| Mild liver disease | Slope before the intervention | 78 | 0.000 | 65 | 92 |
|  | Change in the intercept | -7347 | 0.000 | -8551 | -6143 |
|  | Change in the slope (interaction) | 141 | 0.000 | 65 | 218 |
|  | Intersection | 4662 | 0.000 | 4295 | 5028 |
|  | Linear trend after the intervention | 220 | 0.000 | 144.6282 | 294.3808 |
| Moderate liver disease | Slope before the intervention | 3 | 0.000 | 2 | 5 |
|  | Change in the intercept | -649 | 0.000 | -754 | -544 |
|  | Change in the slope (interaction) | 12 | 0.000 | 6 | 19 |
|  | Intersection | 658 | 0.000 | 608 | 708 |
|  | Linear trend after the intervention | 16 | 0.000 | 9.356879 | 21.92288 |
| Chronic pulmonary disease | Slope before the intervention | 28 | 0.750 | -148 | 205 |
|  | Change in the intercept | -31365 | 0.000 | -38386 | -24344 |
|  | Change in the slope (interaction) | 695 | 0.000 | 357 | 1033 |
|  | Intersection | 44342 | 0.000 | 38911 | 49774 |
|  | Linear trend after the intervention | 723 | 0.000 | 420.4743 | 1026.333 |
| Kidney disease | Slope before the intervention | 160 | 0.000 | 131 | 189 |
|  | Change in the intercept | -8741 | 0.000 | -10545 | -6937 |
|  | Change in the slope (interaction) | 124 | 0.018 | 22 | 227 |
|  | Intersection | 8332 | 0.000 | 7752 | 8913 |
|  | Linear trend after the intervention | 284 | 0.000 | 183.9514 | 384.6524 |
| Rheumatic disease | Slope before the intervention | 47 | 0.000 | 34 | 61 |
|  | Change in the intercept | -7518 | 0.000 | -9005 | -6030 |
|  | Change in the slope (interaction) | 185 | 0.000 | 87 | 283 |
|  | Intersection | 9714 | 0.000 | 9287 | 10142 |
|  | Linear trend after the intervention | 233 | 0.000 | 135.8165 | 329.4262 |
| Peptic ulcer | Slope before the intervention | -3 | 0.174 | -7 | 1 |
|  | Change in the intercept | -1206 | 0.000 | -1453 | -960 |
|  | Change in the slope (interaction) | 41 | 0.000 | 26 | 57 |
|  | Intersection | 2012 | 0.000 | 1871 | 2152 |
|  | Linear trend after the intervention | 38 | 0.000 | 23.38346 | 53.48346 |
| Peripheral vascular disease | Slope before the intervention | 6 | 0.000 | 5 | 7 |
|  | Change in the intercept | -512 | 0.000 | -597 | -426 |
|  | Change in the slope (interaction) | 11 | 0.000 | 5 | 17 |
|  | Intersection | 333 | 0.000 | 302 | 364 |
|  | Linear trend after the intervention | 17 | 0.000 | 10.99521 | 22.23565 |
| Myocardial infarction | Slope before the intervention | 3 | 0.000 | 2 | 5 |
|  | Change in the intercept | -364 | 0.000 | -462 | -267 |
|  | Change in the slope (interaction) | 8 | 0.019 | 1 | 14 |
|  | Intersection | 523 | 0.000 | 478 | 569 |
|  | Linear trend after the intervention | 11 | 0.001 | 4.47767 | 16.67435 |
| Congestive heart failure | Slope before the intervention | 33 | 0.000 | 25 | 41 |
|  | Change in the intercept | -3734 | 0.000 | -4340 | -3129 |
|  | Change in the slope (interaction) | 46 | 0.025 | 6 | 86 |
|  | Intersection | 3692 | 0.000 | 3449 | 3934 |
|  | Linear trend after the intervention | 79 | 0.000 | 40.42742 | 117.9222 |
| Paraplegia and hemiplegia | Slope before the intervention | 9 | 0.000 | 7 | 11 |
|  | Change in the intercept | -1248 | 0.000 | -1465 | -1032 |
|  | Change in the slope (interaction) | 34 | 0.000 | 20 | 49 |
|  | Intersection | 959 | 0.000 | 897 | 1021 |
|  | Linear trend after the intervention | 43 | 0.000 | 28.94408 | 56.9398 |
| AIDS/HIV | Slope before the intervention | 39 | 0.000 | 28 | 50 |
|  | Change in the intercept | -5363 | 0.000 | -6581 | -4144 |
|  | Change in the slope (interaction) | 101 | 0.016 | 19 | 182 |
|  | Intersection | 6190 | 0.000 | 5860 | 6521 |
|  | Linear trend after the intervention | 140 | 0.001 | 59.7266 | 220.0681 |
